# Precise Error Performance of BPSK Modulated Coherent Terahertz Wireless LOS Links with Pointing Errors

**DOI:** 10.3390/e26080706

**Published:** 2024-08-20

**Authors:** Mingbo Niu, Ruihang Ji, Hucheng Wang, Huan Liu

**Affiliations:** Internet of Vehicle^2^ to Road Research Laboratory, School of Energy and Electrical Engineering, Chang’an University, 356 Changda Nan Lu, Xi’an 710018, China; 2023038001@chd.edu.cn (R.J.); 2020132053@chd.edu.cn (H.W.); 2021232020@chd.edu.cn (H.L.)

**Keywords:** atmospheric turbulence, coherent THz, binary phase-shift keying, pointing errors, outage probability

## Abstract

One of the key advantages of terahertz (THz) communication is its potential for energy efficiency, making it an attractive option for green communication systems. Coherent THz transmission technology has recently been explored in the literature. However, there exist few error performance results for such a wireless link employing coherent THz technology. In this paper, we explore a comprehensive terrestrial channel model designed for wireless line-of-sight communication using THz frequencies. The performance of coherent THz links is analyzed, and it is found to be notably affected by two significant factors, atmospheric turbulence and pointing errors. These could occur between the terahertz transmitter and receiver in terrestrial links. The exact and asymptotic solutions are derived for bit error rate and interrupt probability for binary phase-shift keying coherent THz systems, respectively, over log-normal and Gamma–Gamma turbulent channels. The asymptotic outage probability analysis is also performed. It is shown that the presented results offer a precise estimation of coherent THz transmission performance and its link budget.

## 1. Introduction

In light of the rapid development of new generation wireless technology, researchers have begun to explore suitable ranges of the wider frequency spectrum to meet the growing needs globally. The terahertz (THz) band, i.e., typically from 0.1 to 10 THz, has started to attract increasing attention. Due to their higher frequencies, THz systems can utilize smaller antenna sizes and tighter beamforming capabilities, leading to significantly reduced power consumption compared to traditional radio frequency (RF) systems. This property aligns with the development of low-power, sustainable wireless communication infrastructures. Seamless data transfer, unlimited bandwidth, sub-microsecond latency, and ultra-fast THz device will revolutionize communications innovation, providing a new way in which information is transmitted and accessed [1]. One of the key advantages of THz communication in the context of green communications is its potential for low energy consumption. Traditional RF systems, which operate in the MHz to GHz range, face significant challenges in energy efficiency due to power-hungry components and high transmission losses. THz communication, on the other hand, can achieve higher data rates with lower power consumption by leveraging the shorter wavelengths and higher frequencies. This makes THz communication a promising candidate for next-generation green communication networks, where energy efficiency is paramount.

In terms of power consumption, THz communication systems can outperform traditional RF systems by several orders of magnitude. For instance, studies have shown that the power consumption of THz transmitters can be significantly lower than that of RF transmitters at comparable data rates. Moreover, the unique propagation characteristics of THz waves, such as lower interference and higher spatial resolution, enable more efficient use of the available spectrum, further enhancing the energy efficiency of THz communication networks.

THz wireless communication has developed rapidly since the 120 GHz frequency and 10 Gbps communication demonstration system was reported in 2006 [2]. With the advancement of THz communication technology, THz now features faster speeds, higher atmospheric window frequencies, lower power consumption, enhanced integration, and practical applications. Additionally, the data transmission rate of THz communication systems is steadily increasing. A 100 GHz band THz wireless communication system was proposed based on the transmission element surface [3]. The speed of THz communication systems was found to achieve up to 120 Gbps [4]. Using sub-band, airborne THz band communication speed reached 150 Gbps recently [5]. Ref. [6] explored subcarrier-modulated THz wireless systems, analyzing their performance under the log-normal (LN) and Gamma–Gamma (GG) models. It provided a detailed examination of how composite turbulence and pointing errors impact the system performance. However, these studies only considered non-coherent THz links.

More recently, the research based on coherent THz technology is entering an active stage. Coherent THz technology involves the generation and transmission of THz waves using coherent light sources within the THz frequency band. Photoelectric technology was used to generate signals and coherent reception of wireless THz communication [7]. Larger grooves were incorporated into each cycle of the grating, enhancing the transmission grating’s structure as demonstrated in [8], which significantly increased the power output of the coherent THz radiation source. In the development of a reliable THz generator, the light source is driven by a compact accelerator due to the high energy of its pulses and the frequency range between 0.1 THz and 60 THz [9]. Ref. [10] provided explicit expressions for the probability density functions (PDFs), cumulative distribution functions, and secrecy outage probability (SOP) of the end-to-end signal-to-noise ratio (SNR) using Meijer’s G-function. The effectiveness of coherent THz detection using a photoconductive antenna was explored [11], achieving broadband detection capabilities up to 10 THz, thus spanning the entire THz band. Advancements in coherent THz wireless systems, enabled by silicon photonic integrated circuits, have achieved error-free transmission at 1 MHz-THz, with a bit error rate of $10^{-6}$ or lower, and a data rate of 50 Gbps [12]. Comparative studies of two antenna designs, utilizing amplitude modulation and coherent data signals for a wireless link operating near 300 GHz, were detailed [13]. Moreover, the real-time digital coherent transmission of 34 GBd polarization division multiplexing quadrature phase shift keying signals was showcased [14].

Nevertheless, the unpredictable characteristics of the terrestrial channels that coherent THz waves traverse can have devastating effects on the integrity of transmitted data. The propagation of coherent THz signals is not only affected by deterministic attenuations such as molecular absorption and path loss but is also affected by random atmospheric turbulence, especially in outdoor coherent THz signal propagation [15]. Among these factors, the random variation of signal irradiance caused by atmospheric turbulence is a main cause of the performance degradation in coherent THz wireless systems. In order to combat and alleviate the problem, researchers have conducted extensive research on terrestrial turbulent channels and proposed different channel models [16]. However, the random behavior of pointing errors between the transmitter and receivers is another major factor affecting the overall performance of wireless THz links, which needs to be considered for channel model [17]. However, there exist few results for coherent THz wireless link performance considering pointing errors. We comment that, as a quasi-optical wave, the pointing error model in free space optical communication can serve as a valuable reference for addressing THz dislocation fading [18,19,20].

To bridge the aforementioned research gap, in this work, the bit error rate (BER) performance, asymptotic BER performance, and outage probability of a coherent THz systems with pointing errors are investigated. The high-precision convergent sequence BER for LN fading and the approximate sequence BER for GG channels are obtained. The loss of the signal-to-noise ratio (SNR) caused by pointing error is formulated, and the influence of building swaying on the THz links is theoretically solved. Additional insights into coherent THz wireless links are provided through our asymptotic studies.

The rest of this paper is organized as follows. Section 2 formulates the system model for a coherent THz communication link experiencing atmospheric turbulent channels with pointing error. In Section 3, the exact and asymptotic BERs for LN and GG turbulent channels are derived. Section 4 provides an analysis of the outage probability under both weak and strong turbulence conditions. Numerical results and discussions are presented in Section 5. Finally, Section 6 concludes the paper.

## 2. System and Channel Models

### 2.1. System Model

**Figure 1:**
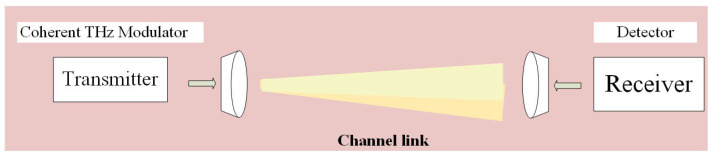
Block diagram of a coherent THz link.

The coherent THz communication system physically combines the received THz wave with the local THz wave to recover signal information from the electric field. This system can provide good background noise suppression and provide better spatial and frequency selectivity. A basic coherent THz link is shown in Figure 1, we assume that both the transmitting and receiving ends are equipped with a high-directional THz transmitter and receiver. After propagating through the atmospheric turbulence channel, the THz signal at the output of the coherent receiver can be modeled as

(1) y=hx+n

where *x* represents the transmitted signal, *h* denotes the channel state, *y* is the synthesized electrical signal at the receiver, and *n* indicates additive white Gaussian noise (AWGN).

It is noted that the pointing error and atmospheric attenuation are independent. Therefore, the channel gain *h*, which comprehensively represents the various interactions—such as path loss, absorption, scattering, and reflection—that THz waves undergo while propagating through the medium from the transmitter to the receiver, can be expressed as $h=h_{l}h_{p}h_{a}$, where $h_{l}$ represents the path loss, remaining constant under specific weather conditions and link distances. The term $h_{p}$ denotes the pointing error loss factor, while $h_{a}$ indicates the atmospheric attenuation loss factor. We observe that $h_{l}$ is deterministic, whereas both $h_{a}$ and $h_{p}$ are random variables (RVs).

### 2.2. Statistical Models

Statistical models play a crucial role in understanding and predicting the behavior of transmitted signals under various environmental conditions. This section delves into three significant aspects of these models: deterministic attenuations, turbulence models, and pointing errors. Each subsection provides a comprehensive analysis of the factors affecting the signal integrity and the mathematical frameworks used to quantify these effects.

#### 2.2.1. Deterministic Attenuations

Wireless propagation at THz frequencies also suffers relatively high attenuation due to interactions between gas molecules and THz waves. The attenuation of THz wave due to molecular absorption has been widely studied. We choose the ITU-R model, which can perform the molecular absorption effect well, especially in the low frequency band of THz frequency (0.1 to 1 THz). The 0.1–1 THz band offers a balance between bandwidth availability and manageable atmospheric attenuation, making it highly suitable for short- to medium-range communication scenarios.

Even if there is no additional attenuation along the wave propagating path, the received power at the receiver will decrease quadratically with the increase in distance. This inevitable loss, named free space path loss (FSPL), always exists and can be calculated using the Friis formula [21] as

(2) $$L_{\mathrm{FS}}=32.4+20\log\left(f\right)+20\log\left(d\right)-G_{\mathrm{TX}}-G_{\mathrm{RX}}$$

where *f* is the frequency in MHz, *d* represents the distance between the transmitter and receiver in kilometers (km), and $G_{TX}$ and $G_{RX}$ denote the antenna gains for the transmitter and receiver, respectively. We use the Beer–Lambert law [22] to describe the loss over the propagation path due to atmospheric attenuation.

(3) $$h_{l}\left(z\right)=\frac{P(z)}{P(0)}=\exp\left(-\sigma z\right)$$

where P(0) is the transmission power, P(z) is the power at a distance of *z* (km), and σ (1/km) is the attenuation coefficient. This parameter can also be measured physically in the atmosphere.

#### 2.2.2. Turbulence Models

In order to analyze the impact of turbulence-induced fading, we present statistical models describing atmospheric fading. For weakly turbulent conditions, one considers a LN fading model to characterize the fading of the atmosphere. Its probability distribution function is as follows [23]:

(4) $$f_{h_{a,\mathrm{LN}}}\left(h_{a}\right)=\frac{1}{2h_{a}\sqrt{2\pi \delta_{X}^{2}}}\exp\left(-\frac{{\left(\ln h_{a}+2\delta_{X}^{2}\right)}^{2}}{8\delta_{X}^{2}}\right)$$

where $\delta_{X}^{2}$ is the log-amplitude variance approximately equal to $\delta_{R}^{2}/4$, where $\delta_{R}^{2}$ is the widely used Rytov variance in the turbulence models which is expressed as $\delta_{R}^{2}=1.23C_{n}^{2}k^{7/6}z^{11/6}$. Additionally, $C_{n}^{2}$ represents the refractive index structure parameter of the atmosphere, *z* denotes the path length, and v=2π/λ is the THz wave number, where λ is the wavelength. For moderate to strong turbulence, the GG model is used widely, and its elaborate PDF is given as [16]

(5) $$\begin{array}{c} f_{h_{a,\mathrm{GG}}}\left(h_{a}\right)=\frac{2{\left(\alpha_{\mathrm{GG}}\beta_{\mathrm{GG}}\right)}^{\frac{\alpha_{\mathrm{GG}}+\beta_{\mathrm{GG}}}{2}}}{\Gamma \left(\alpha_{\mathrm{GG}}\right)\Gamma \left(\beta_{\mathrm{GG}}\right)}{\left(h_{a}\right)}^{\frac{\alpha_{\mathrm{GG}}+\beta_{\mathrm{GG}}}{2}-1}\times K_{\alpha_{\mathrm{GG}}-\beta_{\mathrm{GG}}}\left(2\sqrt{\alpha_{\mathrm{GG}}\beta_{\mathrm{GG}}h_{a}}\right) \end{array}$$

where Γ(·) represents the Gamma function, and $K_{\alpha -\beta }\left(\cdot \right)$ denotes the modified Bessel function of the second kind with an order of $\alpha_{\mathrm{GG}}-\beta_{\mathrm{GG}}$. In the context of plane wave propagation, which is a suitable approximation for long-distance transmissions using reflector antennas, the effective parameters $\alpha_{\mathrm{GG}}$ and $\beta_{\mathrm{GG}}$, associated with small and large eddies in a turbulent medium, are defined as follows [24]:

(6) $$\begin{array}{cc} \; & \alpha_{\mathrm{GG}}={\left[\exp\left(\frac{0.49\sigma_{R}^{2}}{{\left(1+0.65d^{2}+1.11\sigma_{R}^{12/5}\right)}^{7/6}}\right)-1\right]}^{-1} \end{array}$$

(7) $$\begin{array}{cc} \; & \beta_{\mathrm{GG}}={\left[\exp\left(\frac{0.51\sigma_{R}^{2}{\left(1+0.69\sigma_{R}^{12/5}\right)}^{-5/6}}{\left(1+0.9d^{2}+0.62d^{2}\sigma_{R}^{12/5}\right)}\right)-1\right]}^{-1} \end{array}$$

where the parameter *d* is defined as $d=\sqrt{kD^{2}/4z}$, in which *z* is the path distance, *k* is the wavenumber, and *D* is the receiver aperture diameter.

#### 2.2.3. Pointing Errors

**Figure 2:**
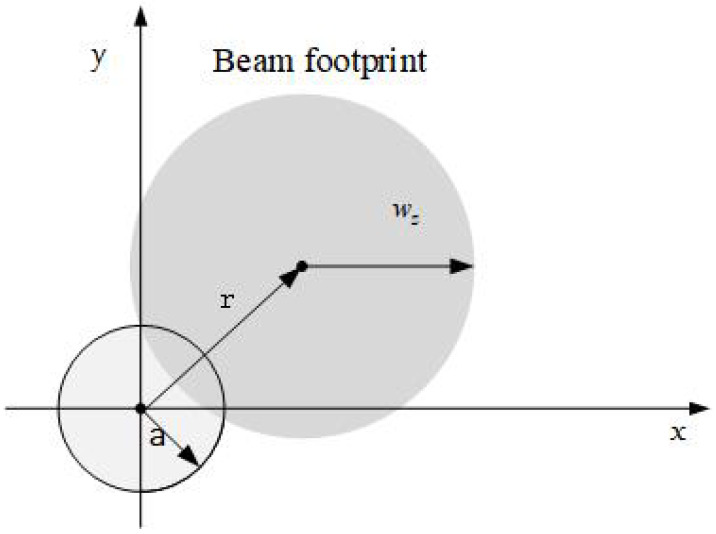
Detector and beam footprint with misalignment on the detector plane.

In line-of-sight (LOS) wireless coherent THz communication links, the loss of pointing error due to misalignment is another important factor to determine the performance and reliability of the link as illustrated in Figure 2.

As shown in Figure 2, consider a receiver (RX) with a circular detection beam that propagates a distance *z* from the transmitter to a circular detector with an aperture radius *a*. The distance *r* represents the instantaneous radial displacement between the center of the detector and the beam’s centroid. The fraction of power collected by the receiver, due to pointing errors, can be approximated by [25]

(8) $$h_{p}\left(r;z\right)\approx A_{0}\exp\left(\frac{-2r^{2}}{w_{z,eq}^{2}}\right)$$

where $A_{0}$ is the fraction of the collected power at r=0, which is defined as $A_{0}={\left[\mathrm{erf}\left(v\right)\right]}^{2}$, and the equivalent beam width $w_{z,\mathrm{eq}}$ is defined as $w_{z,\mathrm{eq}}^{2}=w_{z}^{2}\sqrt{\pi }\frac{\mathrm{erf}(v)}{2v\exp(-v^{2})}$. In this case, $v=\sqrt{\pi }d/\left(\sqrt{2}w_{z}\right)$ represents the ratio of the aperture radius to the beam width. The parameter *d* signifies the receiver aperture’s radius, while $w_{z}$ denotes the beam waist at distance *z*. For a Gaussian beam, the beam waist $w_{z}$ is defined as $w_{z}=w_{0}\sqrt{1+{\left(\frac{z\lambda }{\pi w_{0}^{2}}\right)}^{2}}$, where $w_{0}$ is the beam waist at z=0. Consequently, the PDF of $h_{p}$ can be derived as follows:

(9) $$f_{h_{p}}\left(h_{p}\right)=\frac{\gamma^{2}}{A_{0}^{\gamma^{2}}}h_{p}^{\gamma^{2}-1},\;0\leq h_{p}\leq A_{0}$$

where $\gamma =w_{z,eq}/2\sigma_{S}$ represents the ratio of the equivalent beam width to the jitter standard deviation, quantifying the severity of the pointing error effect.

### 2.3. Channel Statistical Model

The probability distribution of the composite coherent THz wireless channel $h=h_{l}h_{a}h_{p}$ can be expressed as

(10) $$f_{h}\left(h\right)=\int f_{h|h_{a}\left(h|h_{a}\right)}f_{h_{a}}\left(h_{a}\right)dh_{a}$$

Because $h_{l}$ is a fixed parameter that serves as a scaling factor, the conditional distribution can be written as $f_{h|h_{a}}\left(h|h_{a}\right)=\frac{1}{h_{l}h_{a}}f_{h_{p}}\left(\frac{h}{h_{a}h_{l}}\right)$, and the composite PDF for the three distributions of LN and GG can be calculated. We derive the average BER of LN fading with pointing error by substituting Equations (4) and (9) into Equation (10). The composite PDF can be found as

(11) $$f_{e,\mathrm{LN}}\left(h\right)=\frac{\gamma^{2}\exp\left(u_{a}\right)}{2{\left(A_{0}h_{1}\right)}^{\gamma^{2}}}h^{\gamma^{2}-1}\mathrm{erfc}{\left(\frac{\ln\left(\frac{h}{A_{0}h_{i}}\right)+\mu_{b}}{\sqrt{8}\delta_{X}}\right)}^{n}$$

where $\mu_{b}=2\sigma_{X}^{2}\left(1+2\gamma^{2}\right),\mu_{a}=2\sigma_{X}^{2}\gamma^{2}\left(1+\gamma^{2}\right)$.

Similarly, for the GG channel model of Equations (5) and (9) into Equation (10), the composite PDF of the channel can be written as

(12) $$\begin{array}{c} f_{h_{a,\mathrm{GG}}}\left(h\right)=\frac{2\gamma^{2}{\left(\alpha \beta \right)}^{\frac{\alpha +\beta }{2}}h^{\gamma^{2}-1}}{{\left(A_{0}h_{1}\right)}^{\alpha^{2}}\Gamma \left(\alpha \right)\Gamma \left(\beta \right)}\times \int_{\frac{h}{A_{0}h_{0}}}^{\infty }h_{a}^{\frac{\alpha +\beta }{2}-1-\gamma^{2}}K_{\alpha -\beta }\left(2\sqrt{\alpha \beta h_{a}}\right)dh_{a} \end{array}$$

## 3. Bit Error Rate Performance

For the wireless coherent THz systems, the SNR can be expressed as $Y=\bar{\gamma }h$, where $\bar{\gamma }$ is the average SNR, and *h* is the channel gain. Based on the system model outlined in Section 2, the BER for the BPSK modulation is given by

(13) $$P_{e}\left(e|h\right)=Q\left(\sqrt{2\bar{\gamma }h}\right)=\frac{1}{2}\mathrm{erfc}\left(\sqrt{\bar{\gamma }h}\right)$$

The average BER can be expressed as

(14) $$P_{e}=\int_{0}^{\infty }P_{e}\left(e|h\right)f_{h}\left(h\right)dh$$

The average BER can be reformulated by substituting Equation (11) into Equation (14) as follows:

(15) $$\begin{array}{cc} \; & P_{e,\mathrm{LN}}=\frac{\gamma^{2}\exp\left(\mu_{a}\right)}{4{\left(A_{0}h_{1}\right)}^{a^{2}}}\int_{0}^{\infty }h^{\gamma^{2}-1}\mathrm{erfc}\left(\frac{\ln\left(\frac{\Lambda }{A_{0}h_{i}C}\right)+\mu_{b}}{\sqrt{8}\delta_{X}}\right)\mathrm{erfc}\left(\sqrt{\bar{\gamma }h}\right)dh \end{array}$$

By applying a variable transformation, Equation (15) can be written as

(16) $$\begin{array}{c} P_{e,\mathrm{LN}}=\frac{\gamma^{2}\mu_{c}}{4}\exp\left(\mu_{a}-a^{2}\mu_{b}\right)\int_{-\infty }^{\infty }\exp\left(\gamma^{2}\mu_{c}x\right)\mathrm{erfc}\left(\sqrt{\bar{\gamma }A_{0}h_{t}\mu_{c}\exp\left(\mu_{c}x-\mu_{b}\right)}\right)\mathrm{erfc}\left(x\right)\;dx \end{array}$$

By introducing an auxiliary parameter *B* and dividing the integration range in Equation (16) into [−∞,B] and [B,∞], it is possible to derive

(17) $$\begin{array}{cc} P_{e,\mathrm{LN}}= & \frac{\gamma^{2}\mu_{c}}{4}\exp\left(\mu_{a}-a^{2}\mu_{b}\right)\int_{-\infty }^{B}\exp\left(\gamma^{2}\mu_{c}x\right)\\ \; & \times \mathrm{erfc}\left(\sqrt{\bar{\gamma }A_{0}h_{l}\mu_{c}\exp\left(\mu_{c}x-\mu_{b}\right)}\right)\mathrm{erfc}\left(x\right)\;dx+R_{B} \end{array}$$

where $R_{B}$ represents the error in the approximation, which is defined as

(18) $$\begin{array}{c} R_{B}=\frac{\gamma^{2}\mu_{c}}{4}\exp\left(\mu_{a}-\gamma^{2}\mu_{b}\right)\int_{B}^{\infty }\exp\left(\gamma^{2}\mu_{c}x\right)\mathrm{erfc}\left(\sqrt{\bar{\gamma }A_{0}h_{1}\mu_{c}\exp\left(\mu_{c}x-\mu_{b}\right)}\right)\mathrm{erfc}\left(x\right)\;dx. \end{array}$$

The upper bound of the approximation error $R_{B}$ is proved in reference [26]. $R_{B}$ can be quantified under various system parameters, allowing us to calculate the corresponding values of *B*. The findings indicate that the approximation error $R_{B}$ diminishes quickly as *B* grows. By adjusting the value of *B*, the approximation error can be minimized to an arbitrary level. Consequently, the average BER can be approximated as

(19) $$\begin{array}{cc} P_{e,\mathrm{LN}}\approx {\tilde{P}}_{e,\mathrm{LN}}= & \frac{\gamma^{2}\mu_{c}}{4}\exp\left(\mu_{a}-\gamma^{2}\mu_{b}\right)\int_{-\infty }^{B}\exp\left(\gamma^{2}\mu_{c}x\right)\\ \; & \times \mathrm{erfc}\left(\sqrt{\bar{\gamma }A_{0}h_{l}\mu_{c}\exp\left(\mu_{c}x-\mu_{b}\right)}\right)\mathrm{erfc}\left(x\right)\;dx \end{array}$$

In obtaining Equation (19), we use a series expansion of the complementary error function ([27], Equation (06.27.06.0002.01)) and an integral identity ([27], Equation (06.27.21.0011.01)) as

(20) $$\mathrm{erfc}\left(z\right)=\left(-\frac{2}{\sqrt{\pi }}\sum_{k=0}^{\infty }\frac{{\left(-1\right)}^{k}z^{2k+1}}{k!(2k+1)}\right)$$

and

(21) $$\begin{array}{c} \int \exp\left(bz\right)\exp\left(cz\right)\;dz=\frac{1}{b}\left[\exp\left(bz\right)\mathrm{erfc}\left(az\right)-\exp\left(\frac{b^{2}}{4a^{2}}\right)\mathrm{erf}\left(\frac{b}{2a}-az\right)\right] \end{array}$$

where

(22) $$\begin{array}{cc} S_{j}= & \exp\left(\left(2\gamma^{2}+2k+1\right)\mu_{c}B/2\right)\mathrm{erfc}\left(B\right)-\exp\left({\left(2\gamma^{2}+2k+1\right)}^{2}\mu_{c}^{2}/16\right)\\ \; & \times erf(\left(2\gamma^{2}+2k+1\right)\mu_{c}/4-B). \end{array}$$

and

(23) $$\begin{array}{cc} {\tilde{P}}_{e,\mathrm{LN}}= & \frac{\gamma^{2}\mu_{c}\exp\left(\mu_{a}-\mu_{b}^{2}\gamma^{2}\right)}{4}\{\frac{1}{a^{2}\mu_{c}}\left[\exp\left(\gamma^{2}\mu_{c}B\right)\mathrm{erfc}\left(B\right)-\exp\left(\frac{\gamma^{2}\mu_{c}}{4}\right)erf\left(\frac{\gamma^{2}\mu_{c}}{2}-B\right)\right]\\ \; & -\frac{4}{\sqrt{\pi }}\sum_{k=0}^{\infty }\frac{{\left(-1\right)}^{k}{\left(\bar{\gamma }A_{0}h_{l}\mu_{c}\right)}^{\frac{2k+1}{2}}}{k!\left(2k+1\right)\left(2a^{2}+2k+1\right)\mu_{c}}\exp\left(-\frac{\mu_{b}\left(2k+1\right)}{2}\right)S_{j}\}. \end{array}$$

In regions with asymptotically high SNR, the error rates can be approximated by $P_{e}^{\infty }={\left(G_{c}\cdot \bar{\gamma }\right)}^{-G_{d}}$ [28]. Here, $G_{D}$ is the diversity order, which indicates how quickly the BER decreases as SNR increases, and $G_{C}$ is the coding gain, which affects the BER curve’s shift in SNR compared to the benchmark curve ${\left(\bar{\gamma }\right)}^{-G_{d}}$. Analyzing the asymptotic BER helps understand system performance in high SNR regions and the factors that limit performance in terrestrial coherent THz links. From the behavior of the PDF of the instantaneous SNR near the origin, the diversity order and coding gain can be derived. The PDF of the channel gain can be expanded into a power series, given by $\lim_{h\to 0}f_{h}\left(h\right)=ah^{t}+g_{t}\left(h\right)$, where $g_{t}\left(h\right)$ satisfies $\lim_{h\to 0}\frac{g_{t}\left(h\right)}{h^{t}}=0$. Thus, in our system, the diversity order and coding gain can be determined, respectively, as

(24) $$G_{d}=\frac{t+1}{2}$$

and

(25) $$G_{c}={\left(\frac{2^{\frac{t-3}{2}}a\Gamma \left(\frac{t}{2}+1\right)}{\sqrt{\pi }\left(\frac{t+1}{2}\right)}\right)}^{-\frac{2}{t+1}}.$$

Here, *t* is a parameter related to the behavior of the PDF near the origin, and *a* is a constant. The diversity order $G_{d}$ indicates how the BER improves with increasing SNR, while the coding gain $G_{c}$ provides a measure of how efficiently the coding scheme utilizes the available SNR. Consequently, for composite LN fading with pointing errors, performing a limit operation on Equation (11) yields the PDF of the channel gain *h* near the origin as derived in Section 2:

(26) $$\lim_{h\to 0}f_{\mathrm{LN}}\left(h\right)=\frac{\gamma^{2}}{\left(A_{0}h_{l}\right)}\exp\left(\mu_{a}\right)h^{\gamma^{2}-1}+g_{\gamma^{2}-1}\left(h\right).$$

The asymptotic BER can then be obtained from $P_{e}^{\infty }={\left(G_{c}\cdot \bar{\gamma }\right)}^{-Gd}$. Using Equations (21)–(23), the diversity order and coding gain can be determined, then the asymptotic BER can be expressed as

(27) $$P_{e,\mathrm{LN}}^{\infty }=\frac{2^{\frac{\gamma^{2}}{2}-1}\Gamma \left(\frac{\gamma^{2}}{2}+\frac{1}{2}\right)\exp\left(\mu_{a}\right)}{\sqrt{\pi }{\left(A_{0}h_{l}\right)}^{a^{2}}}{\left(\bar{\gamma }\right)}^{-\frac{\gamma^{2}}{2}}.$$

For the GG channel model, using Equations (12)–(14), the average BER can be written as

(28) $$\begin{array}{c} P_{e,\mathrm{GG}}=\int_{0}^{\infty }\left\{\frac{1}{2}\mathrm{erfc}\left(\sqrt{\bar{\gamma }h}\right)\frac{2\gamma^{2}{\left(\alpha \beta \right)}^{\frac{\alpha +\beta }{2}}}{{\left(A_{0}h_{l}\right)}^{\gamma^{2}}\Gamma \left(\alpha \right)\Gamma \left(\beta \right)}h^{\gamma^{2}-1}\int_{\frac{h}{A_{0}h_{l}}}^{\infty }h_{\alpha }^{\frac{\alpha +\beta }{2}-1-\alpha^{2}}K_{\alpha +\beta }\left(2\sqrt{\alpha \beta h_{\alpha }}\right)dh_{\alpha }\right\}dh. \end{array}$$

The composite PDF of the GG model can be approximated [29] by finite series as follows:

(29) $$\begin{array}{cc} \; & f_{\mathrm{GG}}\left(h\right)\approx \sum_{j=0}^{J}\left\{\frac{1}{j!}{\left(\frac{\alpha \beta }{A_{0}h_{l}}\right)}^{j}\left(\nu_{j}\left(\alpha ,\beta \right)h^{\beta -1+j}-\nu_{j}\left(\beta ,\alpha \right)h^{\alpha -1+j}\right)\right\} \end{array}$$

where $J=\left\lfloor \gamma^{2}-\alpha \right\rfloor$ and

(30) $$\begin{array}{c} \nu_{j}\left(\alpha ,\beta \right)=\frac{\gamma^{2}\pi {\left(\frac{\alpha \beta }{A_{0}h_{l}}\right)}^{\beta }\sin^{-1}\left(\left(\alpha -\beta \right)\pi \right)}{\Gamma \left(\alpha \right)\Gamma \left(\beta \right)\Gamma \left(j-\left(\alpha -\beta \right)+1\right)|-(\beta -\alpha^{2}+j)|}. \end{array}$$

It should be noted that although Equation (29) is an approximation of the composite PDF for GG fading with pointing error, it is valid under the condition $\gamma^{2}>\alpha$ and converges rapidly as the value of *J* increases. Utilizing an integral identity ([27], Equation (6.281.1)),

(31) $$\int_{0}^{\infty }\left(1-\Phi \left(px\right)\right)x^{2q-1}dx=\frac{\Gamma (q+\frac{1}{2})}{2\sqrt{\pi }qp^{2q}}$$

one can obtain an approximate BER with finite series as follows:

(32) $$\begin{array}{cc} P_{e,\mathrm{GG}}=\sum_{j=0}^{J}\{ & \frac{1}{j!}{\left(\frac{\alpha \beta }{A_{0}h_{l}}\right)}^{j}\left(\nu_{j}\left(\alpha ,\beta \right)\frac{\Gamma (\beta +j+\frac{1}{2})}{2\sqrt{\pi }\left(\beta +j\right){\bar{\gamma }}^{\beta +j}}-\nu_{j}\left(\beta ,\alpha \right)\frac{\Gamma (\alpha +j+\frac{1}{2})}{2\sqrt{\pi }\left(\alpha +j\right){\bar{\gamma }}^{\alpha +j}}\right)\}. \end{array}$$

We notice that Equation (32) is obtained without additional approximation in Equation (27). The PDF power series expansion near the origin for GG composite fading channels is derived as follows:

(33) $$\lim_{I\to 0}=\nu_{0}\left(\alpha ,\beta \right)I^{\beta -1}+g_{\beta -1}\left(h\right)$$

where $v_{0}\left(\alpha ,\beta \right)$ is defined as in Equation (29), and it is assumed that $\gamma^{2}>\alpha$. Consequently, the asymptotic BER can be found as follows:

(34) $$\begin{array}{c} P_{e,\mathrm{GG}}^{\infty }=\frac{2^{\frac{\beta }{2}-1}\Gamma \left(\frac{\beta }{2}+\frac{1}{2}\right){\left(\frac{\alpha \beta }{A_{0}h_{1}}\right)}^{\beta }}{\Gamma (\alpha )\Gamma (\beta )\sin((\alpha -\beta )\pi )\Gamma (-(\alpha -\beta )+1)}\frac{1}{|\gamma^{2}-\beta |\beta }\Gamma \left(\frac{\beta +1}{2}\right)\sqrt{\pi }\gamma^{2}{\bar{\gamma }}^{-\frac{\beta }{2}}. \end{array}$$

It is interesting to find that when $\gamma^{2}>\alpha$, the diversity order is $G_{d}=\beta /2$. This suggests that in high average SNR regions, the GG fading effect has a greater impact on BER performance than the pointing error effect.

## 4. Outage Probability Analysis

Besides BER, outage probability is another important performance index of coherent THz wireless links. The outage probability is the chance that the instantaneous capacity is insufficient to meet the necessary data rate [30]. Thus, the outage probability of a given coherent THz link can be expressed as

(35) $$P_{\mathrm{outage}}\left(\Lambda \right)=\mathrm{Pr}\left(Y<\Lambda \right)=\int_{0}^{\Lambda }f_{Y}\left(Y\right)dY$$

where Pr(·) represents the probability of outage occurrence, Λ is the predefined outage threshold, and $f_{Y}\left(Y\right)$ is the PDF of the instantaneous SNR. With our derived composite PDF models, the outage probability under a specific transmission condition can be calculated. For weak turbulence, substituting the relationship $Y=\bar{\gamma }h$ and Equation (12) into Equation (35), one obtains the outage probability for LN channels as

(36) $$\begin{array}{cc} P_{\mathrm{out},\mathrm{LN}}= & \frac{1}{2}[\exp\left(\mu_{c}\right)\mathrm{erfc}\left(\frac{\ln\left(\frac{\Lambda }{A_{0}h_{1}\bar{\gamma }}\right)+\mu_{b}}{\mu_{c}}\right)-\exp\left(\mu_{a}-\gamma^{2}\mu_{b}+\frac{\gamma^{4}\mu_{c}^{2}}{4}\right)\\ \; & \times \mathrm{erf}\left(\frac{\gamma^{2}\mu_{c}}{2}-\frac{\ln\left(\frac{\Lambda }{A_{0}h_{1}\bar{\gamma }}\right)+\mu_{b}}{\mu_{c}}\right)]. \end{array}$$

## 5. Numerical Results and Discussion

**Figure 3:**
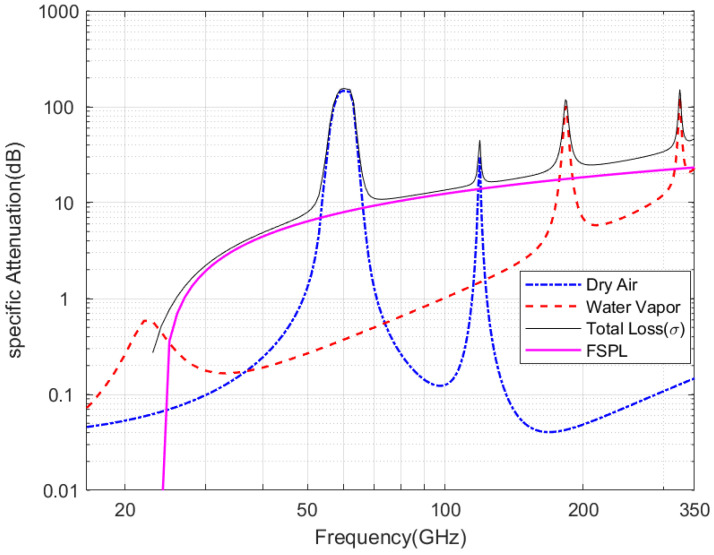
Atmospheric decay due to molecular absorption and FSPL losses in the frequency ranges of 1 GHz to 350 GHz.

In this section, we present numerical studies of the coherent THz links on turbulent conditions from weak to strong, as well as the different propagation path distances. The effects of varying turbulence strengths (from weak to strong) composited with pointing errors on coherent THz links are considered, and the error performance of such links is studied. Figure 3 shows atmospheric attenuation at frequencies up to 1 THz due to dry air and moisture under clear weather conditions using the ITU-R model. In addition, at a propagation path length of 1000 m, the FSPL with transmitter and receiver gain of 60 dBi is shown in Figure 3. The total deterministic loss $h_{l}$ is the sum of the atmospheric loss and FSPL.

Figure 3 gives the attenuation of Earth’s atmosphere in the frequency range of 1 GHz to 350 GHz at sea level, the FSPL link path of 1 km at a pressure of 1013 hPa, temperature of 20 °C, and water vapor density of 7.5 g/m^3^. We select a 300 GHz fixed frequency link and use an emerging high-gain LOS parabolic offset reflector at the transmitter and receiver.

The basic system parameters of the THz link are summarized in Table 1. It is worth noting that since the THz transmitter receiver is affected by the half-power beam width, the corresponding beam width $\omega_{z}$ on the receiver side is much larger than the receiver aperture diameter. Without loss of generality, one can assume 2.1 mrad beam divergence and 1 mrad pointing error. The performance of coherent THz wireless links is influenced by weather conditions, which can be characterized by refractive index structure parameter $C_{n}^{2}$ and attenuation $h_{l}$.

**Figure 4:**
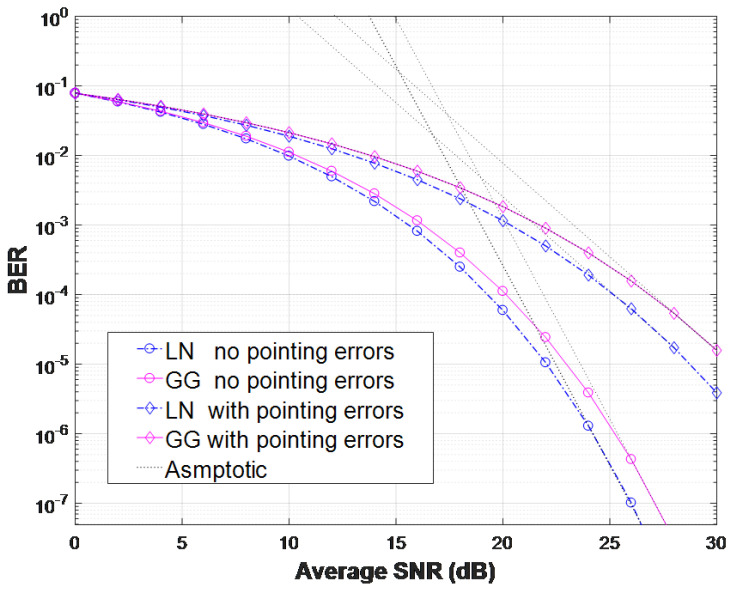
BER performance of coherent THz links for two different turbulent channels with z=200 m.

Given that the LN channel model is appropriate only for weak turbulence conditions, a relatively short link distance of z=200 m is considered to compare the BER performance between the LN and GG composite channels as depicted in Figure 4. In a separate analysis of the GG composite channel, BERs for weak ($\delta_{R}^{2}$ = 0.35), moderate ($\delta_{R}^{2}$ = 1.26), and strong ($\delta_{R}^{2}$ = 24.15) turbulence conditions are presented for a THz link distance of z=1000 m.

In Figure 4, the pointing error effects on the BER performance of coherent THz links are clearly shown. The BER performance decreases with the addition of a pointing error. Though not shown in the figures, our results have been confirmed by Monte Carlo simulations.

Excellent agreement is found between our series (with J=30) and the exact BER solutions, which demonstrate the high accuracy of our approximate BER solutions. In addition, the exact BER and the asymptotic BER show a matching consistency at high SNR regions. Compared to the GG composite model, the LN composite model represents lower turbulence and therefore has better BER performance at the same transmission distance. From Figure 4, a less than 1 dB SNR difference can be found between LN and GG composite models at a BER of $10^{-6}$.

**Figure 5:**
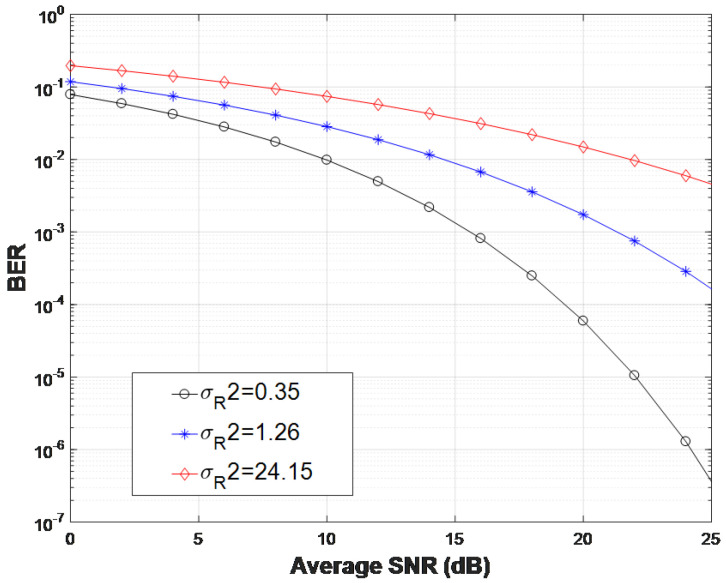
BER of coherent THz links for different turbulence intensities GG channel with pointing errors for a distance z=1000 m.

In Figure 5, BERs for weak ($\delta_{R}^{2}$ = 0.35), moderate ($\delta_{R}^{2}$ = 1.26), and strong ($\delta_{R}^{2}$ = 24.15) composite GG channels are given for coherent BPSK THz links. It is evident that BER performance degrades significantly as the turbulence strength increases. For instance, at an average SNR of 20 dB, the BER is around $10^{-2}$ under strong turbulence, while it improves to approximately $10^{-4}$ under weak turbulence.

**Figure 6:**
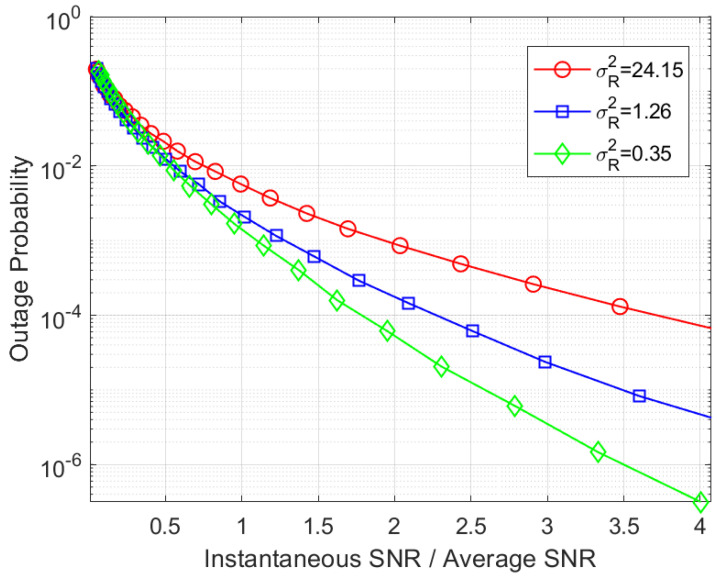
The outage probability of coherent THz links over the GG fading channel with pointing errors.

Therefore, Figure 6 presents the outage probability for conditions ranging from weak to strong turbulence with Λ = 3 dB. Our series solutions closely match the exact outage curves with J=30 in practical SNR regimes.

## 6. Conclusions

In this work, the performance of coherent THz links was investigated. Both closed-form solutions for BER and outage probability were obtained for such THz wireless links. Asymptotic solutions were also derived and revealed some interesting insights into the coherent THz wireless links. The presented results can be used to estimate coherent THz links performance effectively and rapidly over LN and GG composited channels. Numerical studies demonstrated that the derived closed-form solutions are highly accurate for coherent THz link system performance estimation. In conclusion, our exploration of coherent THz transmission technology provides valuable insights into its performance under various channel conditions. Incorporating green communication principles underscores the potential of THz systems in achieving low-power, efficient, and sustainable wireless communication.

## Figures and Tables

**Table 1:** Basic system parameters with values.

Parameter	Value
Frequency	0.3 THz
Receiver diameter D(2a)	300 mm for 60 dBi
Noise standard deviation $\left(\sigma_{n}\right)$	$10^{-7}$ A/Hz
Link distance	*z* m
Half-angle beam divergence	2.1 mrad
Corresponding beam radius $\left(\omega_{z}\right)$	2.1 mrad × *z*
Jitter angle	1 mrad
Corresponding jitter standard deviation $\left(\sigma_{s}\right)$	1 mrad × *z*

## Data Availability

The original contributions presented in the study are included in the article.

## References

[1] Chen Z., Ma X., Zhang B., Zhang Y., Niu Z., Kuang N., Chen W., Li L., Li S. (2019). A survey on terahertz communications. China Commun..

[2] Wang C.-X., Huang J., Wang H., Gao X., You X., Hao Y. (2020). 6G oriented wireless communication channel characteristics analysis and modeling. arXiv.

[3] Yang H., Zheng S., Zhang H., Li N., Shen D., He T., Yang Z., Lyu Z., Yu X. (2023). A THz-OAM wireless communication system based on transmissive metasurface. IEEE Trans. Antennas Propag..

[4] Kopyt P., Salski B., Zagrajek P., Janczak D., Sloma M., Jakubowska M., Olszewska-Placha M., Gwarek W. (2016). Electric properties of graphene-based conductive layers from DC up to terahertz range. IEEE Trans. Terahertz Sci. Technol..

[5] Kokkoniemi J., Jornet J.M., Petrov V., Koucheryavy Y., Juntti M. (2021). Channel modeling and performance analysis of airplane-satellite terahertz band communications. IEEE Trans. Veh. Technol..

[6] Niu M., Ji R., Zhang P., Miah M.S., Li Y. (2024). Error performance and capacity of subcarrier BPSK modulated THz wireless systems with pointing errors. Opt. Express.

[7] Harter T., Weber M., Muehlbrandt S., Wolf S., Kemal J., Boes F., Nellen S., Goebel T., Giesekus J., Zwick T. Wireless THz communications using optoelectronic techniques for signal generation and coherent reception. Proceedings of the 2017 Conference on Lasers and Electro-Optics (CLEO).

[8] Xu X., Hu M., Zhang Z., Zhang X. Enhanced coherent THz radiation by dual groove grating Smith-Purcell effect. Proceedings of the 2020 45th International Conference on Infrared, Millimeter, and Terahertz Waves (IRMMW-THz).

[9] Zhang K., Kang Y., Liu T., Wang Z., Feng C., Fang W., Zhao Z. (2021). A compact accelerator-based light source for high-power, full-bandwidth tunable coherent THz generation. Appl. Sci..

[10] Wang D., Wu M., Wei Z., Yu K., Min L., Mumtaz S. (2023). Uplink secrecy performance of RIS-based RF/FSO three-dimension heterogeneous networks. IEEE Trans. Wirel. Commun..

[11] Liu S., Shou X., Nahata A. (2011). Coherent detection of multiband terahertz radiation using a surface plasmon-polariton based photoconductive antenna. IEEE Trans. Terahertz Sci. Technol..

[12] Lee W., Han S., Moon S.-R., Park J., Yoo S., Park H., Lee J.K., Yu K., Cho S.H. (2022). Coherent terahertz wireless communication using dual-parallel MZM-based silicon photonic integrated circuits. Opt. Express.

[13] Nellen S., Lauck S., Peytavit E., Szriftgiser P., Schell M., Ducournau G., Globisch C. (2022). Coherent wireless link at 300 GHz with 160 Gbit/s enabled by a photonic transmitter. J. Light. Technol..

[14] Castro C., Elschner R., Merkle T., Schubert C., Freund R. 100 Gb/s real-time transmission over a THz wireless fiber extender using a digital-coherent optical modem. Proceedings of the Optical Fiber Communication Conference.

[15] Han C., Chen Y. (2018). Propagation modeling for wireless communications in the terahertz band. IEEE Commun. Mag..

[16] Badarneh O.S. (2022). Performance analysis of terahertz communications in random fog conditions with misalignment. IEEE Wirel. Commun. Lett..

[17] Boulogeorgos A.-A.A., Papasotiriou E.N., Alexiou A. (2019). Analytical performance assessment of THz wireless systems. IEEE Access.

[18] Jamshed M.A., Nauman A., Abbasi M.A.B., Kim S.W. (2020). Antenna selection and designing for THz applications: Suitability and performance evaluation: A survey. IEEE Access.

[19] Stratidakis G., Papasotiriou E.N., Konstantinis H., Boulogeorgos A.-A.A., Alexiou A. Relay-based blockage and antenna misalignment mitigation in THz wireless communications. Proceedings of the 2020 2nd 6G Wireless Summit (6G SUMMIT).

[20] Papasotiriou E.N., Boulogeorgos A.-A.A., Alexiou A. (2020). Performance analysis of THz wireless systems in the presence of antenna misalignment and phase noise. IEEE Commun. Lett..

[21] Schneider T., Wiatrek A., Preußer S., Grigat M., Braun R.-P. (2012). Link budget analysis for terahertz fixed wireless links. IEEE Trans. Terahertz Sci. Technol..

[22] Rodger A. (2018). Absorption Spectroscopy, the Beer-Lambert Law, and Transition Polarizations.

[23] Forin D.M., Incerti G., Beleffi G.M., Tosi G., Teixeira A.L.J., Costa L.N., De Brito P.S., Geiger B., Leitgeb E., Nadeem F. Free Space Optical Technologies. https://pdfs.semanticscholar.org/a6c0/b7c629965c616fe98ff1633c53669de90e35.pdf.

[24] Sun J., Huang P., Yao Z. (2018). Diversity reception technology in coherent optical communication over gamma-gamma atmospheric turbulence channel. Acta Opt. Sin..

[25] Yang F., Cheng J., Tsiftsis T.A. (2014). Free-space optical communication with nonzero boresight pointing errors. IEEE Trans. Commun..

[26] Ma X., Chen Z., Chen W., Li Z., Chi Y., Han C., Li S. (2020). Joint channel estimation and data rate maximization for intelligent reflecting surface assisted terahertz MIMO communication systems. IEEE Access.

[27] (2001). The Wolfram Functions Site. http://functions.wolfram.com/.

[28] Wang Z., Giannakis G.B. (2003). A simple and general parameterization quantifying performance in fading channels. IEEE Trans. Commun..

[29] Bhatnagar M.R., Ghassemlooy Z. (2016). Performance analysis of gamma–gamma fading FSO MIMO links with pointing errors. J. Lightwave Technol..

[30] Petrov V., Moltchanov D., Koucheryavy Y., Jornet J.M. (2020). Capacity and outage of terahertz communications with user micro-mobility and beam misalignment. IEEE Trans. Veh. Technol..

